# Exposure to Gestational Intermittent Hypoxia Does Not Impair the Metabolic Function or Accelerate the Biological Ageing Process of Offspring of Either Sex

**DOI:** 10.1111/jsr.70245

**Published:** 2025-11-14

**Authors:** Esther Valverde‐Perez, Margarida B. Almeida, Joana F. Sacramento, Elena Olea, Jesus Prieto‐Lloret, Asuncion Rocher, Silvia V. Conde

**Affiliations:** ^1^ Instituto de Biomedicina y Genética Molecular (IBGM) Consejo Superior de Investigaciones Científicas – Universidad de Valladolid Valladolid Spain; ^2^ Departamento de Bioquímica y Biología Molecular y Fisiología, Facultad de Medicina Universidad de Valladolid Valladolid Spain; ^3^ INOVA4Health, NOVA Medical School, Faculdade de Ciências Médicas Universidade NOVA de Lisboa Lisboa Portugal; ^4^ Departamento de Enfermería, Facultad de Enfermeria Universidad de Valladolid Valladolid Spain

**Keywords:** chronic intermittent hypoxia, hepatic function, life‐course, metabolic dysfunction, offspring, pregnancy, sex

## Abstract

Obstructive sleep apnea (OSA), marked by intermittent hypoxia, is associated with obesity, type 2 diabetes and metabolic associated fatty liver disease. In pregnancy, it remains underdiagnosed despite links to gestational diabetes, hypertension, and foetal growth restriction. Intermittent hypoxia may alter foetal programming and increase the risk of long‐term metabolic issues in offspring. This study evaluates the effects of gestational OSA on offspring metabolic function, focusing on weight gain, glucose homeostasis, insulin sensitivity, hepatic glucose metabolism, inflammation and oxidative stress. Experiments were performed on pregnant female Wistar rats submitted to a chronic intermittent hypoxia (CIH) protocol during the last 2 weeks of pregnancy. Offspring were evaluated for body weight, glucose tolerance and insulin sensitivity at 1, 3, and 12 months of age. Liver western blot analysis was performed to assess markers of glucose metabolism (glucokinase, pyruvate kinase and glucose‐6‐phosphatase), inflammation (NF‐kB, IL‐1R, IL‐6R, TNF‐ɑR and NRLP3) and antioxidant enzymes (catalase, SOD‐1 and iNOS). CIH did not modify body weight, glucose tolerance and insulin sensitivity at 1, 3 and 12 months of age, except for a transient increase in glucose intolerance observed in 3‐month‐old females, which was attenuated by 12 months. Moreover, no evidence was found of modifications caused by gestational CIH on markers of hepatic glucose metabolism, inflammation or antioxidant defence. However, there was a gradual increase in inflammation with age. No sexual dimorphism was observed. Overall, these findings suggest that gestational CIH does not predispose offspring to long‐term metabolic dysfunction later in life and does not affect biological ageing, regardless of sex.

## 1 Introduction

Obstructive sleep apnea (OSA) is a prevalent sleep‐related breathing disorder characterised by recurrent episodes of airflow reduction (hypopnea) or complete cessation (apnea) due to upper airway collapse during sleep (Sateia 2014; Jordan et al. 2014; Lévy et al. 2015). These episodes trigger chronic intermittent hypoxia (CIH) and frequent arousals, leading to sleep fragmentation (Sateia 2014; Jordan et al. 2014; Lévy et al. 2015). OSA is increasingly recognised as an important contributor to systemic metabolic dysfunction, being strongly associated with insulin resistance, glucose intolerance, dyslipidemia, and an increased risk of type 2 diabetes (Almendros et al. 2022; Bonsignore et al. 2013; Li et al. 2018; Reutrakul and Mokhlesi 2017). The underlying mechanisms are multifactorial, involving sympathetic overactivation, oxidative stress, systemic inflammation, and hormonal imbalance (Almendros et al. 2022; Bonsignore et al. 2013).

Recent studies have emphasised the role of the liver as a key organ mediating OSA‐associated metabolic disturbances. CIH has been shown to impair hepatic insulin signalling, increase gluconeogenesis, and promote lipid accumulation in the liver, ultimately contributing to hepatic steatosis and systemic insulin resistance (Almendros et al. 2022; Isaza et al. 2020; Hazlehurst et al. 2022). These hepatic alterations involve oxidative stress, mitochondrial dysfunction and inflammatory pathways activation (Fernandes et al. 2023; Ji et al. 2023; Savransky et al. 2007; Wang et al. 2018).

During pregnancy, physiological and anatomical changes—such as body weight gain, fluid retention, and hormonal fluctuations—enhance the susceptibility to develop OSA (Lin et al. 2008; Maniaci et al. 2024; Tayade and Toshniwal 2022). Maternal OSA has been associated with increased risks of gestational diabetes, preeclampsia, hypertension, and foetal growth restriction (Maniaci et al. 2024; Tayade and Toshniwal 2022). Despite this, gestational OSA remains underdiagnosed and undertreated.

More recently, research has begun to uncover the impact of maternal CIH on foetal programming, raising concerns that in utero exposure to intermittent hypoxia may predispose offspring to long‐term metabolic and cardiovascular dysfunction.

The potential mechanisms by which gestational OSA affects the foetus include both indirect effects—through maternal systemic alterations such as increased inflammation and oxidative stress—and direct effects of hypoxia on the foetus via placental oxygen exchange (Alonso‐Fernández et al. 2021; Song et al. 2024). While some studies report markers of foetal hypoxia and placental dysfunction in pregnancies affected by OSA (Badran, Abuyassin, et al. 2019; Valverde‐Pérez et al. 2023), others suggest a protective buffering effect of the placenta, particularly in response to mild or transient hypoxic episodes (Ravishankar et al. 2015; Arthuis et al. 2017; Almendros et al. 2019).

Despite some controversy, a growing body of literature indicates that gestational OSA and CIH can have lasting effects on offspring health (for a review see Valverde‐Pérez et al. 2024). Human and animal studies have reported associations between in utero exposure to CIH and adverse outcomes, including low birth weight, metabolic dysregulation, cognitive impairments, and altered neurodevelopment (Badran, Yassin, et al. 2019; Iqbal and Ciriello 2013; Khalyfa et al. 2017; Song et al. 2022; Vanderplow et al. 2022; Wongkitikamjorn et al. 2023). Animal models, in particular, have demonstrated sex‐specific responses, with male offspring more prone to developing obesity, insulin resistance, increased adiposity, systemic inflammation, and vascular dysfunction (Badran, Yassin, et al. 2019; Iqbal and Ciriello 2013) while female offspring appear relatively protected (Badran, Yassin, et al. 2019)—potentially due to the influence of sex hormones such as oestrogen.

Nevertheless, the precise mechanisms and long‐term metabolic consequences of gestational CIH, particularly regarding hepatic function and sex differences, remain unclear. In this study, we use a rat model of gestational CIH to investigate its impact on offspring metabolic health across different stages of postnatal life. We focus on key indicators of systemic metabolism (body weight, fasting glycaemia, glucose tolerance and insulin sensitivity), as well as hepatic glucose metabolism, oxidative stress and inflammation. Additionally, we examine whether sex modulates these outcomes.

## 2 Materials and Methods

### 2.1 Animals and Experimental Protocol

Experiments were conducted on 3‐month‐old breeding female Wistar rats that were crossed with male Wistar rats. The presence of a copulation plug was an indicator of the first day of gestation. All mothers were maintained under normoxic conditions during the first 7 days of gestation. After, they were randomly divided into two groups: the control group (CTL; *n* = 7) which continued to be exposed to normoxic air and the experimental group (CIH; *n* = 6), which was exposed to CIH for the remaining 14 days of gestation. This period was chosen because it represents a critical window of foetal growth and metabolic programming in rodents, partially corresponding to the third trimester in humans (Christoforou and Sferruzzi‐Perri 2020), when obstructive sleep apnea is most frequently diagnosed or exacerbated in pregnant women (Maniaci et al. 2024). The protocol of IH consisted of cycles of exposure for 40 s to 5% O_2_, then exposure to air for 80 s, repeating this cycle for 8 h per day (08:00–16:00), corresponding to ~30 events per hour, which is equivalent to an apnea–hypopnea index (AHI) in the severe range. To this end, the CIH group was placed in hermetically sealed methacrylate chambers. A gas control delivery system regulated the flow of room air, N_2_ and O_2_ into the customised cages housing the rats (Figure S1). All pregnant rats (CTL and CIH) were housed in the same room in the vivarium of the University of Valladolid, with free access to food and water, under controlled conditions of temperature and humidity. On the last day of gestation (day 21) the IH exposure ceased. The dams were placed in their respective cages to give birth in normoxic conditions. All litters were standardised to eight pups per mother, and offspring from each litter were included across all age groups (1, 3 and 12 months). Pups were weaned at postnatal day 21. At each time point (1, 3 and 12 months of age), a total of 32 animals were evaluated: 8 males and 8 females from each experimental group (CTL and CIH), ensuring sex balance and equal representation of both conditions. The offspring were housed under normoxic conditions, with *ad libitum* access to food and water, controlled temperature and humidity, and a 12‐h light/dark cycle until the time of sacrifice by the administration of a lethal dose of sodium pentobarbital.

**FIGURE 1:**
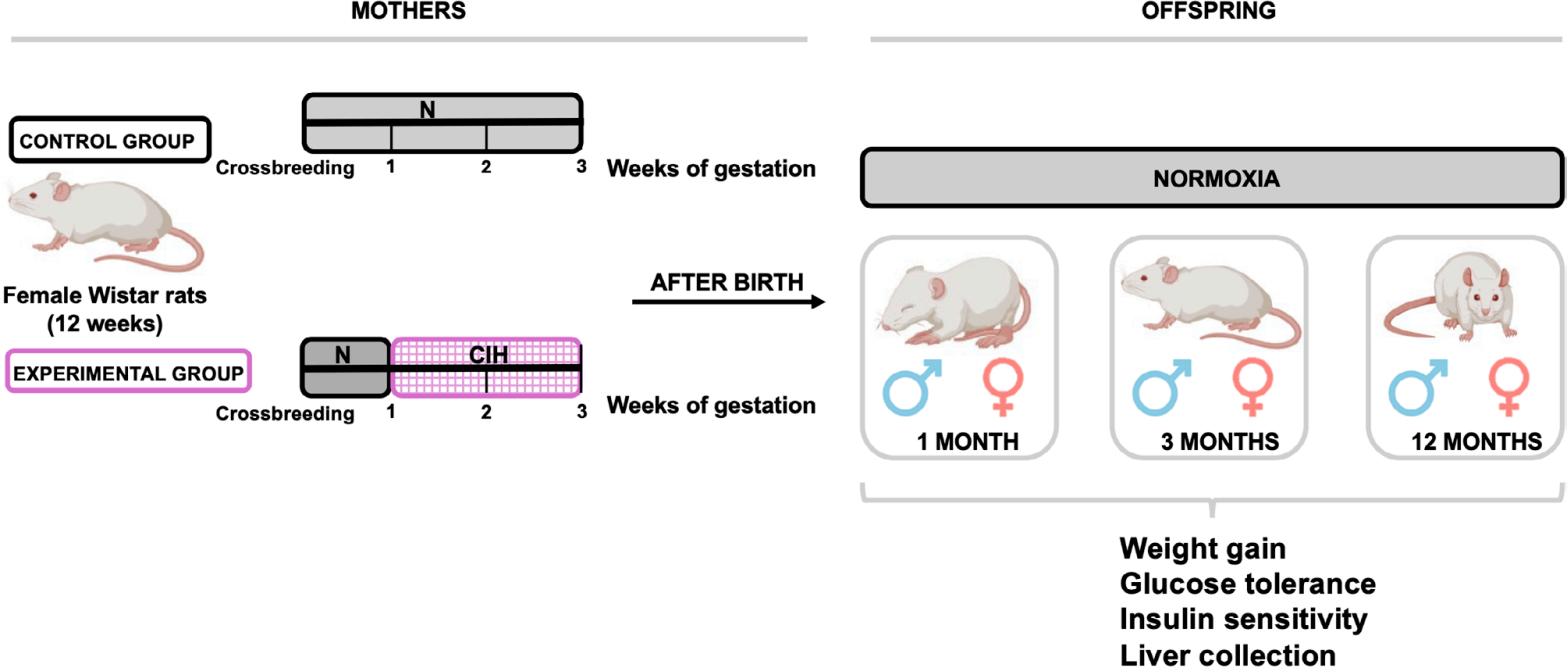
Schematic illustration of the experimental protocol and the assessments of the study. IH, intermittent hypoxia; N, normoxia. ♂, male; ♀, female.

At each age, animals were weighed and evaluated for glucose tolerance and insulin sensitivity. Moreover, blood was collected for the measurement of insulin levels and liver was collected for Western blot analysis at the NOVA Medical School. Figure 1 illustrates the mothers and offspring from various age groups employed throughout this study (Figure 1).

The experimental protocols and procedures designed for this work were approved by the University of Valladolid Institutional Committee for Animal Care and Use (Project Approval Ethical Code: 9601981) and NOVA Medical School, Faculdade de Ciências Médicas. They were conducted following the European Community Council Directive for Protection of Vertebrates Used for Experimental and Other Scientific Ends (2010/63/EU). Sample size calculation was performed at https://www.calculator.net/samplesizecalculator.html and based on our previous experiments using the same protocol of CIH in male rats (Fernandes et al. 2023; Prieto‐Lloret et al. 2021) and in transgenerational studies in mice (Badran, Abuyassin, et al. 2019). For the calculations, we assumed a moderate effect size (Cohen's *f* ≈0.25), a significance level (*α*) of 0.05, and a statistical power (1− *β*) of 0.80 (80%).

### 2.2 Evaluation of Insulin Sensitivity and Glucose Tolerance

Glucose tolerance was evaluated in the offspring at each established time point through an oral glucose tolerance test (OGTT). After fasting for at least 8 h, basal glucose levels were measured in the tail vein using a glucometer (Contour Next, Ascensia Diabetes Care) with the corresponding test strips. Afterward, a glucose solution (0.2 g glucose/mL H_2_O) was administered orally using a syringe fitted with a cannula. The volume of glucose solution administered was adjusted according to the animal's body weight, following a ratio of 1 mL per 100 g of body weight. Blood glucose measurements were taken at 15, 30, 60, 90 and 120 min, following the same procedure used for baseline measurement. The glucose excursion curves were used to calculate the area under the curve.

Insulin sensitivity was assessed through the homeostasis model assessment (HOMA‐IR) index 2–3 days after the glucose challenge/test was performed. To calculate insulin plasma levels, blood was collected after heart puncture into tubes containing EDTA‐K3 (Vacutainer Hemogard). Blood was centrifuged at 1000×*g* for 20 min at 4°C and stored at −80°C until further analysis. Plasma insulin levels were measured using a commercial ELISA kit specific for rats (EZRMI‐13K, Millipore). For HOMA assessment, fasting glycaemia was measured on the same day as insulinemia.

### 2.3 Western Blot Analysis of Proteins Involved in Hepatic Glucose Metabolism, Inflammation and Antioxidant Defence

Liver tissue (50 mg) from offspring was homogenised in 0.5 mL of lysis buffer (100 mM Tris–HCl pH 7.5, 50 mM EDTA, 0.2 M EGTA, 0.27 M sucrose, 1% Triton X‐100, 100 mM NaF, 10 mM Na_2_PO_4_, 0.1 Na_3_VO_4_), supplemented with protease and phosphatase inhibitors. Samples were homogenised using an automatic disperser (T18 Digital Ultra Turrax, IKA, Germany), centrifuged at 13000 rpm for 20 min at 4°C (Eppendorf 5415R), and supernatants were stored at −80°C. Protein concentrations were determined using the BCA assay (MicroBCA Protein Assay Kit, Thermo Scientific). For the Western blot equal amounts of protein (10–20 μg) were denaturated in loading buffer (4× sample buffer with beta‐mercaptoethanol, 1:1) at 99°C for 5 min. Samples (16 μL) and molecular weight markers were loaded on SDS‐PAGE gels (10% (resolving) running and 4% stacking gels) and separated by SDS‐polyacrylamide gel electrophoresis. Proteins were transferred to nitrocellulose membranes (90 min at 400 mA, 4°C) using transfer buffer (25 mM Tris, 190 mM glycine, 20% methanol). Membranes were stained with Ponceau, washed with 0.1% TBS‐T, and blocked for 90 min in 5% non‐fat dry milk in TBS‐T. Membranes were then incubated overnight at 4°C with primary antibodies: rabbit anti‐pyruvate carboxylase (PC‐B) (1:1000, sc‐67021 Santa Cruz Biotechnology, Madrid, Spain), mouse anti‐glucokinase (1:200, sc‐177819 Santa Cruz Biotechnology, Madrid, Spain), rabbit anti‐glucose‐6‐phosphatase‐α (G6Pase) (1:500, sc‐25840 Santa Cruz Biotechnology, Madrid, Spain), goat anti‐catalase (1:500, Ab0245 SicGen, Portugal), rabbit anti‐inducible nitric oxide synthase (iNOS) (1:500, sc‐7271 Santa Cruz Biotechnology, Madrid, Spain), mouse anti‐interleukin 1 receptor (IL‐1R) (1:100, sc‐393998 Santa Cruz Biotechnology, Madrid, Spain), mouse anti‐interleukin 6 receptor (IL‐6R) (1:200, sc‐373708 Santa Cruz Biotechnology, Madrid, Spain), rabbit anti‐NLR family pyrin domain containing 3 (NLRP3) (1:200, MA5‐32255 Invitrogen, Rockford, USA), rabbit anti‐nuclear factor kappa B (NF‐κB) (1:500, sc‐372 Santa Cruz Biotechnology, Madrid, Spain), mouse anti‐superoxide dismutase 1 (SOD‐1) (1:500, sc‐101523 Santa Cruz Biotechnology, Madrid, Spain), and mouse anti‐tumour necrosis factor alpha receptor (TNF‐αR) (1:200, sc‐8436 Santa Cruz Biotechnology, Madrid, Spain). After washing, membranes were incubated with HRP‐conjugated secondary antibodies for 90 min at room temperature: Mouse Anti‐Goat (1:2000, Santa Cruz Biotechnology, Heidelberg, Germany), Goat Anti‐Mouse (1:1000, Bio‐Rad Laboratories, Madrid, Spain), Goat Anti‐Rabbit (1:2000, GE Healthcare, Madrid, Spain). Signal detection was performed using Clarity Western ECL substrate (Bio‐Rad) and imaged with a Chemidoc system (Bio‐Rad). Band intensities were quantified using Image Lab Software (v6.1.0). Protein expression was normalised to calnexin (goat, 1:1000, Ab0041 SicGen, Portugal), an established endoplasmic reticulum marker and has been validated in several studies as a suitable loading control in metabolic and hypoxia‐related contexts (Lakkaraju and van der Goot 2013; Paskevicius et al. 2023), or β‐actin (goat, 1:1000, Ab0145 SicGen, Portugal), using β‐actin as an alternative when calnexin signals were weak.

### 2.4 Statistical Analysis

The data was analysed using GraphPad Prism Software 8.0.2 (GraphPad Software Inc., USA). The results were expressed as mean values with SEM. Normality was assessed using the Shapiro–Wilk test, and all data met the assumptions for ANOVA. The significance of the differences was determined by the three‐way analysis of variance (ANOVA) with Tukey's multicomparison test. Differences were considered significant at *p* < 0.05. a1, a2, a3 and a4 meaning *p* < 0.05, *p* < 0.01, *p* < 0.001 and *p* < 0.0001 respectively when comparing the effect of ageing on animals born from normoxic mothers; b1, b2, b3 and b4 meaning *p* < 0.05, *p* < 0.01, *p* < 0.001 and *p* < 0.0001 respectively when comparing the effect of ageing on animals born from CIH mothers; c1, c2, c3 and c4 meaning *p* < 0.05, *p* < 0.01, *p* < 0.001 and *p* < 0.0001 respectively when comparing the effect of sex on animals born from normoxic mothers; d1, d2, d3 and d4 meaning *p* < 0.05, *p* < 0.01, *p* < 0.001 and *p* < 0.0001 respectively when comparing the effect of sex on animals born from CIH mothers; e1, e2, e3 and e4 meaning *p* < 0.05, *p* < 0.01, *p* < 0.001 and *p* < 0.0001 respectively when comparing the effect of gestational CIH.

## 3 Results

### 3.1 Impact of Gestational Intermittent Hypoxia on Metabolic Function in the Offspring

**FIGURE 2:**
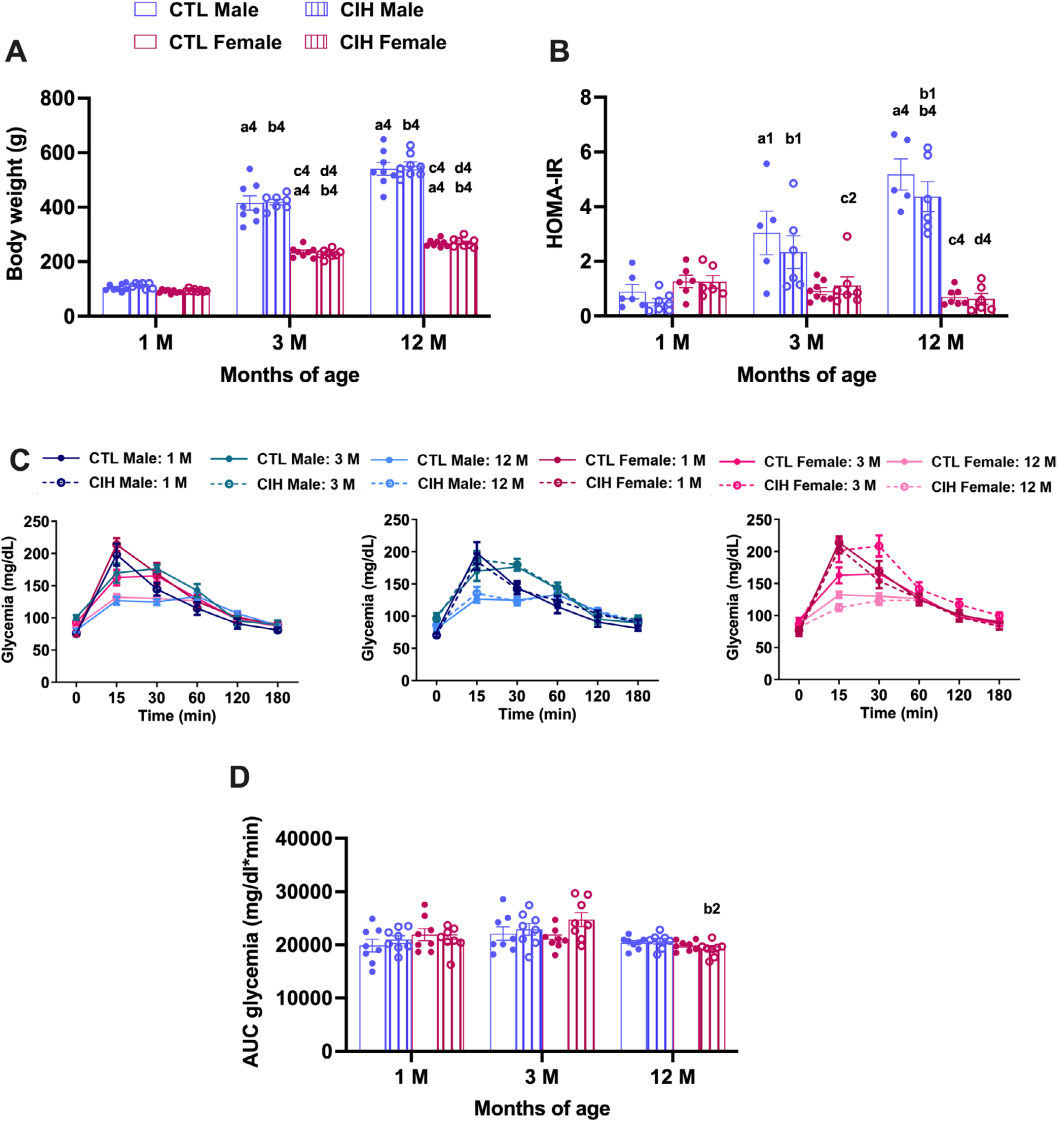
Effect of gestational chronic intermittent hypoxia (CIH) and age on whole‐body metabolic parameters, body weight (A), glucose tolerance (B, C); and insulin sensitivity (D) in the male and female offspring at 1, 3 and 12 months of age. Panel A shows the effect of gestational CIH on body weight in male and female offspring. Panels B and C show respectively, the glucose excursion curves of the oral glucose tolerance test and the correspondent area under the curve (AUC) in male and female offspring. Panel D shows insulin sensitivity evaluated using HOMA‐IR index in male and female offspring. Animal groups: CTL—control (offspring from mothers only exposed to normoxia during pregnancy); CIH—chronic intermittent hypoxia (offspring from mothers exposed to CIH during pregnancy). Animals born from mothers in normoxia are represented by empty bars and from CIH mothers by hatched bars, with blue for males and bordeaux for females. Data are presented as means ± SEM. Three‐way ANOVA with Tukey's multiple comparison tests; a1, and a4 meaning *p* < 0.05, and *p* < 0.0001 respectively when comparing the effect of ageing on animals born from normoxic mothers; b1 and b2 meaning *p* < 0.05, *p* < 0.01, respectively when comparing the effect of ageing on animals born from CIH mothers; c4 meaning *p* < 0.0001 when comparing the effect of sex on animals born from normoxic mothers; d4 meaning *p* < 0.0001 when comparing the effect of sex on animals born from CIH mothers.

Figure 2 depicts the effects of gestational CIH during 2 weeks on metabolic variables in male and female offspring at 1, 3 and 12 months of age. A three‐way ANOVA (sex × gestational condition × age) revealed significant main effects of age (*p* < 0.0001) and sex (*p* < 0.0001) on body weight, with 3‐ and 12‐month‐old animals showing higher values than 1‐month‐old animals, and males weighing more than females. A significant age × sex interaction was also observed (*p* < 0.0001), indicating that the pattern of age‐related changes differed between sexes. Interestingly, the same pattern was observed for insulin resistance, herein assessed as the HOMA. The three‐way ANOVA (sex × gestational condition × age) revealed significant main effects of age (*p* < 0.0001) and sex (*p* < 0.0001), with 3‐ and 12‐month‐old animals showing higher insulin resistance values than 1‐month‐old animals in males but not in females (Figure 2B). In contrast, only age‐related differences (*p* < 0.0001) were observed in glucose tolerance (Figure 2C,D), with an increase in glucose intolerance in the experimental group at 3‐month‐old females (*p* = 0.018) that was attenuated by 12 months of age (Figure 2D). Our data showing that ageing increases fasting insulin levels (Table 1) and insulin resistance in males (Figure 2) are consistent with previous reports (Guarino et al. 2013; Kahn et al. 2006; Kolb et al. 2023). This effect was not observed in females (Figure 2B), possibly because they may still retain circulating sex hormones at 12 months of age, which could confer metabolic protection. Moreover, the increase in insulin resistance in males does not translate into impaired glucose homeostasis, possibly because at 12 months, which corresponds to approximately 37–48 years in humans (https://www.jax.org/research‐and‐faculty/research‐labs/the‐harrison‐lab/gerontology/life‐span‐as‐a‐biomarker), the animals are still able to compensate for higher insulin levels, which may explain the absence of more pronounced age‐related impairments in glucose peaks.

**TABLE 1:** Effect of gestational chronic intermittent hypoxia (CIH) on the levels of fasting glycaemia and insulin in the offspring at 1, 3 and 12 months of age.

Sex	Males			Females		
Age	1	3	12	1	3	12
Glycaemia (mg/dL)						
CTL	75.50 ± 4.36	100.63 ± 3.89^a4^	80.63 ± 2.68	76.50 ± 5.06	92.75 ± 3.63	86.50 ± 2.41
CIH	70.25 ± 2.55	96.00 ± 3.16^b4^	85.62 ± 2.03^b4^	73.00 ± 5.27	85.50 ± 2.14	81.50 ± 2.63
Insulin (ng/mL)						
CTL	4.84 ± 1.39	12.26 ± 2.91	27.15 ± 3.16^a4^	6.29 ± 1.01	4.05 ± 0.60^c4^	3.32 ± 0.55^c4^
CIH	2.87 ± 0.71	9.53 ± 2.16	21.25 ± 2.99^b4^	6.55 ± 1.05	5.27 ± 1.33^d4^	4.48 ± 1.15^b4,d4^

*Note:* Values represent means ± SEM. Three‐way ANOVA with Tukey's multi‐comparison test; a1, a2, a3 and a4 meaning *p* < 0.05, *p* < 0.01, *p* < 0.001 and *p* < 0.0001 respectively when comparing the effect of ageing on animals born from normoxic mothers; b1, b2, b3 and b4 meaning *p* < 0.05, *p* < 0.01, *p* < 0.001 and *p* < 0.0001 respectively when comparing the effect of ageing on animals born from CIH mothers; c1, c2, c3 and c4 meaning *p* < 0.05, *p* < 0.01, *p* < 0.001 and *p* < 0.0001 respectively when comparing the effect of sex on animals born from normoxic mothers; d1, d2, d3 and d4 meaning *p* < 0.05, *p* < 0.01, *p* < 0.001 and *p* < 0.0001 respectively when comparing the effect of sex on animals born from CIH mothers; e1, e2, e3 and e4 meaning *p* < 0.05, *p* < 0.01, *p* < 0.001 and *p* < 0.0001 respectively when comparing the effect of gestational CIH.

No significant main effect of gestational CIH was detected in the three‐way ANOVA, as CIH during gestation did not affect body weight in male or female offspring at any of the ages studied (Figure 2A). Moreover, gestational CIH had no effect on fasting glycaemia (Table 1), on insulin sensitivity (Figure 2B) and on glucose tolerance at 1, 3, or 12 months of age (Figure 2C,D).

**FIGURE 3:**
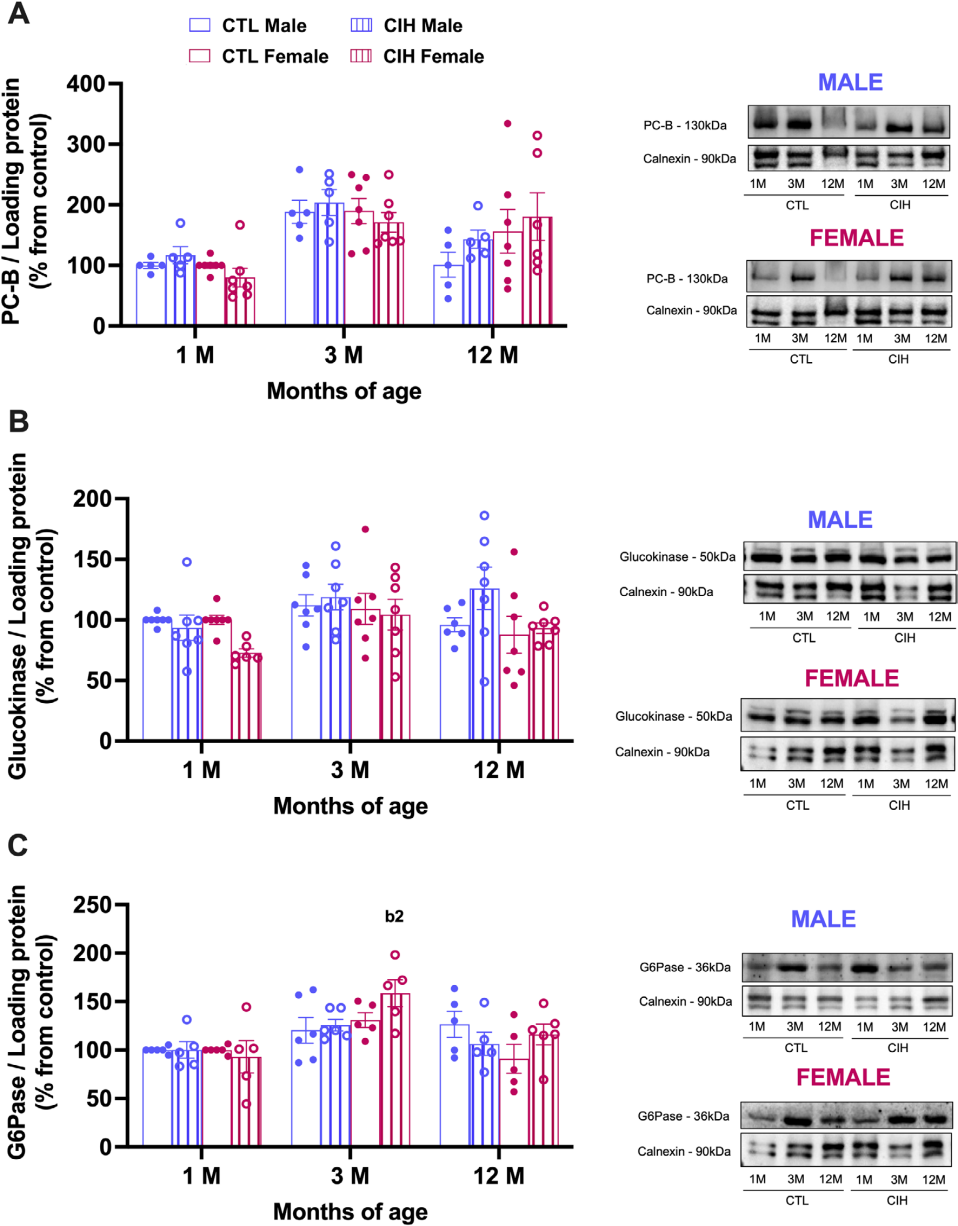
Effects of gestational chronic intermittent hypoxia (CIH) and age on hepatic protein levels involved in glucose metabolism in the offspring. Panels A–C depict respectively the levels of pyruvate carboxylase (PC‐B, 130 kDa), the levels of glucokinase (50 kDa), and the levels of glucose‐6‐phosphatase (G6Pase, 36 kDa) in male and female offspring. Protein levels were normalised to the loading protein calnexin (90 kDa). The representative western blots for each protein are presented at the right side of each graph. Animals born from mothers in normoxia are represented by empty bars and from CIH mothers by hatched bars, with blue for males and bordeaux for females. Data is presented as means ± SEM. Three‐Way ANOVA with Tukey's multicomparison test with b2 meaning *p* < 0.01 when comparing the effect of ageing on animals born from CIH mothers.

Since the liver plays a crucial role in maintaining blood glucose homeostasis (Han et al. 2016), we also evaluated key proteins in these regulatory processes. A three‐way ANOVA (sex × gestational condition × age) revealed significant main effects of age on the levels of PC‐B (*p* < 0.0001), a key enzyme in intermediary metabolism involved in gluconeogenesis, lipogenesis, and TCA cycle replenishment (Figure 3A); glucokinase (*p* = 0.034), which regulates hepatic glucose metabolism and glycogen storage (Figure 3B); and glucose‐6‐phosphatase (*p* < 0.0001), which controls both gluconeogenesis and glycogen breakdown (Figure 3C). Additionally, a significant main effect of sex was observed for glucokinase (*p* = 0.031). Tukey's post hoc analysis revealed no changes at PC‐B or glucokinase (Figure 3A,C). G6Pase levels were increased in 3‐month‐old females born from CIH animals (*p* = 0.0057), an effect not seen at 12 months of age (Figure 3C). No significant main effects of gestational CIH were observed.

### 3.2 Impact of Gestational Intermittent Hypoxia on Hepatic Antioxidant and Pro‐Oxidant Capacity in the Offspring

**FIGURE 4:**
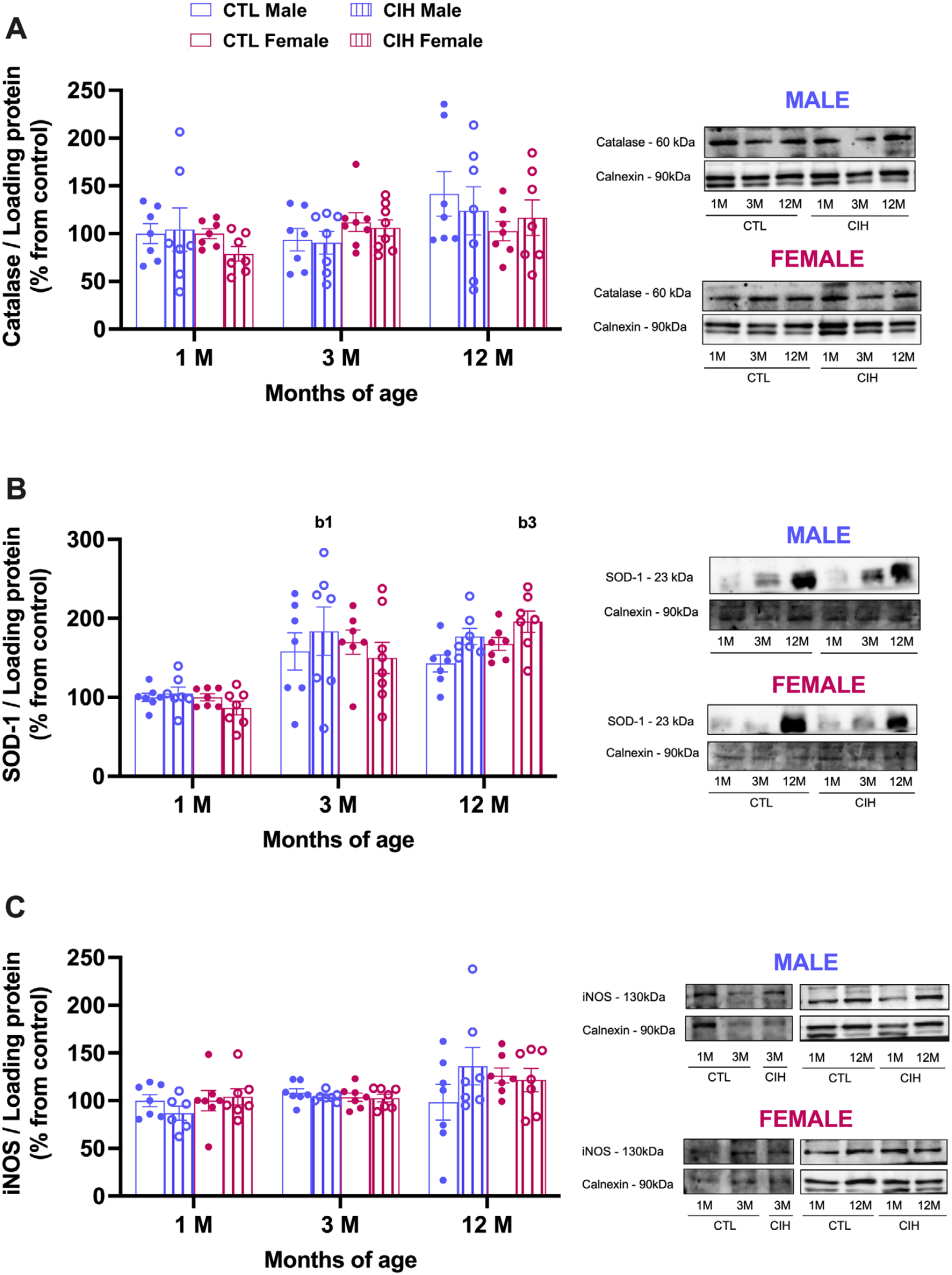
Effects of gestational chronic intermittent hypoxia (CIH) and age on the levels of proteins involved in antioxidant and oxidant capacity in the liver of the offspring. Panels A–C depict respectively the levels of catalase (60 kDa), the levels of SOD‐1 (23 kDa), and the levels of iNOS (130 kDa) in male and female offspring. Protein levels were normalised to the loading protein calnexin (90 kDa). The representative western blots for each protein are presented at theright side of each graph. Animals born from mothers in normoxia are represented by empty bars and from CIH mothers by hatched bars, with blue for males and bordeaux for females. Data is presented as means ± SEM. Three‐way ANOVA with Tukey's multicomparison test b1 and b3 meaning *p* < 0.05, *p* < 0.001, respectively when comparing the effect of ageing on animals born from CIH mothers.

Given that OSA‐associated metabolic dysfunction has been linked to hepatic oxidative stress (Fernandes et al. 2023) we have investigated the impact of gestational CIH on the levels of several antioxidant and pro‐oxidant enzymes in the liver of the offspring (Figure 4). Interestingly, a three‐way ANOVA (sex × gestational condition × age) revealed significant main effects of age for catalase (*p* = 0.0466) (Figure 4A), SOD‐1 (*p* < 0.0001) (Figure 4B) and iNOS (*p* = 0.0085) (Figure 4C), but no significant effects of sex (catalase *p* = 0.4696; SOD‐1 *p* = 0.9538; iNOS = 0.4990) or gestational CIH (catalase *p* = 0.5703; SOD‐1 *p* = 0.2668; iNOS = 0.6035). Post hoc Tukey analysis revealed significant increases only for females born from CIH mothers at 12 months of age (female: CIH 1 month = 86.54 ± 8.57%; CIH 3 month = 149.89 ± 19.85%, *p* = 0.13; CIH 12 month =195.00 ± 13.50%, *p* = 0.0002), but that do not differ from controls, SOD‐(CTL12month = 167.6 ± 8.17%, *p* = 0.978). Altogether, these results suggest a possible upregulation of antioxidant capacity in response to the age‐related rise in pro‐oxidant activity (Rea et al. 2018), with no significant effects of sex or gestational CIH.

### 3.3 Impact of Gestational Intermittent Hypoxia on Hepatic Inflammation Markers in the Offspring

**FIGURE 5:**
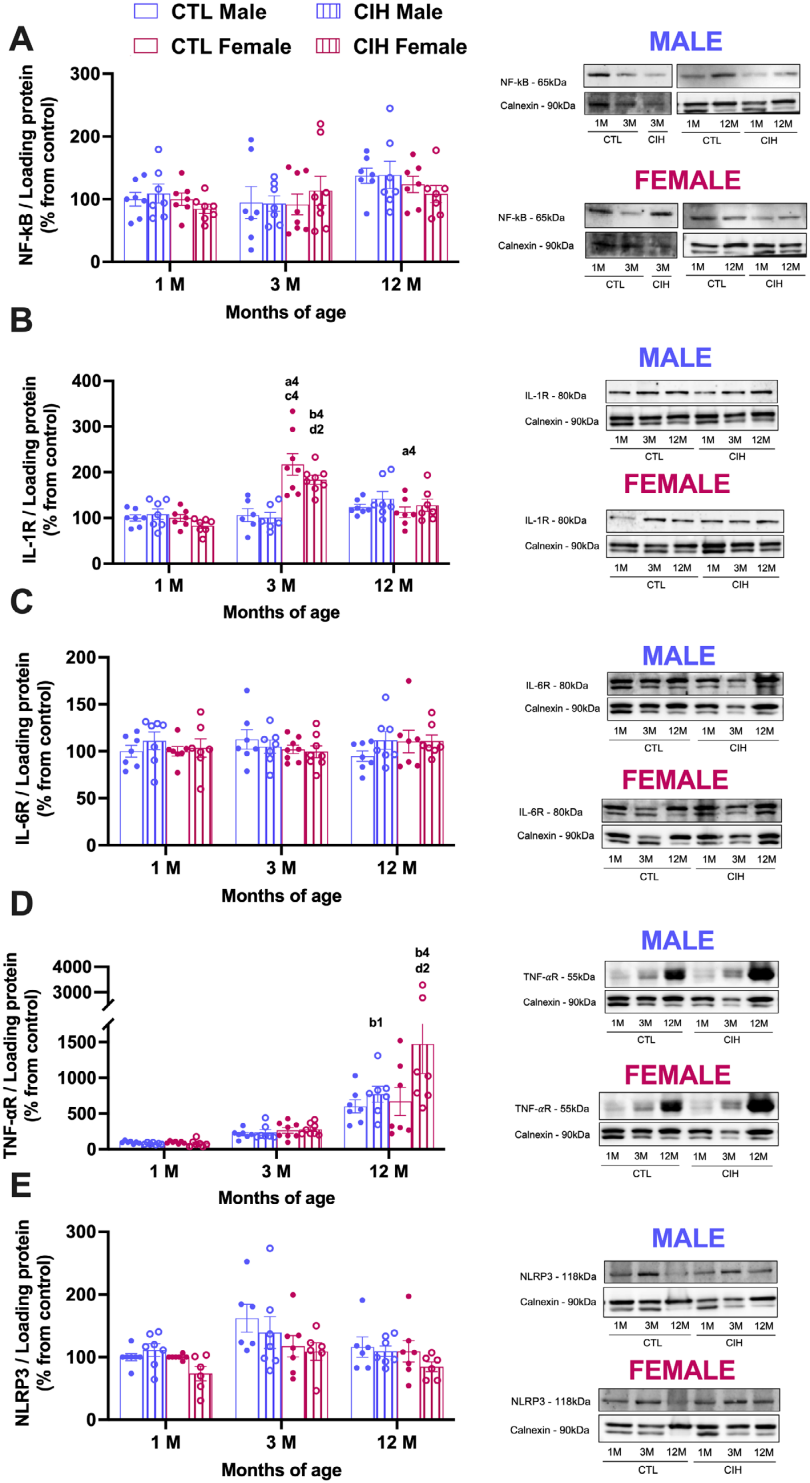
Effects of gestational CIH on the levels of inflammatory markers in the liver of the offspring. Panels A–E depict respectively the levels of NF‐κB (65 kDa), the levels of IL‐6R (80 kDa), the levels of IL‐1R (80 kDa), the levels of TNF‐αR (55 kDa) and the levels of NLRP3 (118 kDa) in male and female offspring. Protein levels were normalised to the loading protein calnexin (90 kDa). The representative western blots for each protein are presented at the right side of each graph. Animals born from mothers in normoxia are represented by empty bars and from CIH mothers by hatched bars, with blue for males and bordeaux for females. Data is presented as means ± SEM. Three‐way ANOVA with Tukey's multicomparison test with a4 meaning *p* < 0.0001 when comparing the effect of ageing on animals born from normoxic mothers; b1 and b4 meaning *p* < 0.05, *p* < 0.0001, respectively when comparing the effect of ageing on animals born from CIH mothers; c4 meaning *p* < 0.0001 when comparing the effect of sex on animals born from normoxic mothers and d2 meaning *p* < 0.01 when comparing the effect of sex on animals born from CIH mothers.

Knowing that OSA is linked to hepatic inflammation and liver disease (Almendros et al. 2022; Aron‐Wisnewsky et al. 2012) and that experimental CIH is associated with liver inflammation (Fernandes et al. 2023; Savransky et al. 2007), we investigated the impact of gestational CIH on some inflammatory markers in the liver of the offspring (Figure 5). A three‐way ANOVA (sex × gestational condition × age) revealed no main effects of the exposure to gestational CIH in the hepatic levels of NF‐kB (*p* = 0.999) and NLRP3 (*p* = 0.1312), as well as in the levels of the receptors of IL‐6 (*p* = 0.4306), IL‐1 (*p* = 0.7424) and TNF‐α (*p* = 0.1907) in the offspring (Figure 5A–E). Nonetheless, several main effects were observed related to age (NF‐kB *p* = 0.021; IL‐1R *p* < 0.0001; IL‐6R *p* = 0.842; TNF‐α *p* < 0.0001 and NLRP3 *p* = 0.0028) and sex (NF‐kB *p* = 0.366; IL‐1R *p* < 0.0001; IL‐6R *p* = 0.753; TNF‐α *p* = 0.071 and NLRP3 *p* = 0.0061) of the offspring. However, although, the levels of NF‐kB showed a tendency to increase in both sexes at 12 months of age, particularly in males (CTL 1 month = 100.0 ± 10.95%; CTL 12 month = 137.5 ± 12.10%, *p* = 0.997); females (CTL 1 month = 100.0 ± 9.77%; CTL 12 month = 123.6 ± 13.07%, *p* = 0.997), Tukey post hoc analysis did not find any significant difference (Figure 5A). Interestingly, while no differences were found in IL‐6R with age (Figure 5B), suggesting an absence of effect of this interleukin on hepatic inflammatory processes associated with age, the levels of IL‐1R and TNF‐αR in the liver were altered during ageing in females and in both sexes, respectively. Tukey's post hoc analysis revealed no effects in the levels of IL‐1R in males (Figure 5B). In contrast, in females, the levels significantly increased at 3 months of age by 117.3% (*p* < 0.0001) and 83.7% (*p* = 0.0006) in controls and CIH animals, respectively, an effect attenuated at 12 months of age (Figure 5B, Controls: CTL 3 months = 217.28 ± 23.65%; CTL 12 months = 112.35 ± 11.41%, *p* < 0.0001; gestational CIH: CIH 3 months = 183.66 ± 9.67%; CIH 12 months = 127.43 ± 13.31%, *p* = 0.075). These findings indicate a transient sexual dimorphism in IL‐1R levels at 3 months of age.

Regarding TNF‐αR, there was a main effect of age (*p* < 0.0001), but with no significant sex differences (*p* = 0.1907) and no effects of gestational CIH (Figure 5D). Interestingly, while there was an increase of 115.8% and 501.1% at 3 and 12 months of age in male control offspring (Figure 5D, control: CTL 1 month = 100 ± 7.25%; CTL 3 months = 215.80 ± 24.23%, *p* > 0.999; CTL 12 months = 601.06 ± 90.02, *p* = 0.3195) and of 166.2% and 571.1% in female control offspring (Figure 5D, control: CTL 1 month = 100 ± 6.57%; CTL 3 months = 266.16 ± 39.58%, *p* = 0.998; CTL 12 months = 671.80 ± 197.1%, *p* = 0.183), Tukey post hoc analysis did not find any significant differences. TNF‐αR levels in the animals born from mothers exposed to CIH also increased by 197.1% (*p* = 0.999) and 863.1% (*p* = 0.03) in 3 and 12 months old males, respectively, and by 281.01% (*p* = 0.996) and 1471.63% (< 0.0001) in females at the same ages (Figure 5D). Altogether, these data suggest that there is a progressive inflammatory process in the liver with ageing, in which TNF‐α plays an active role.

## 4 Discussion

**FIGURE 6:**
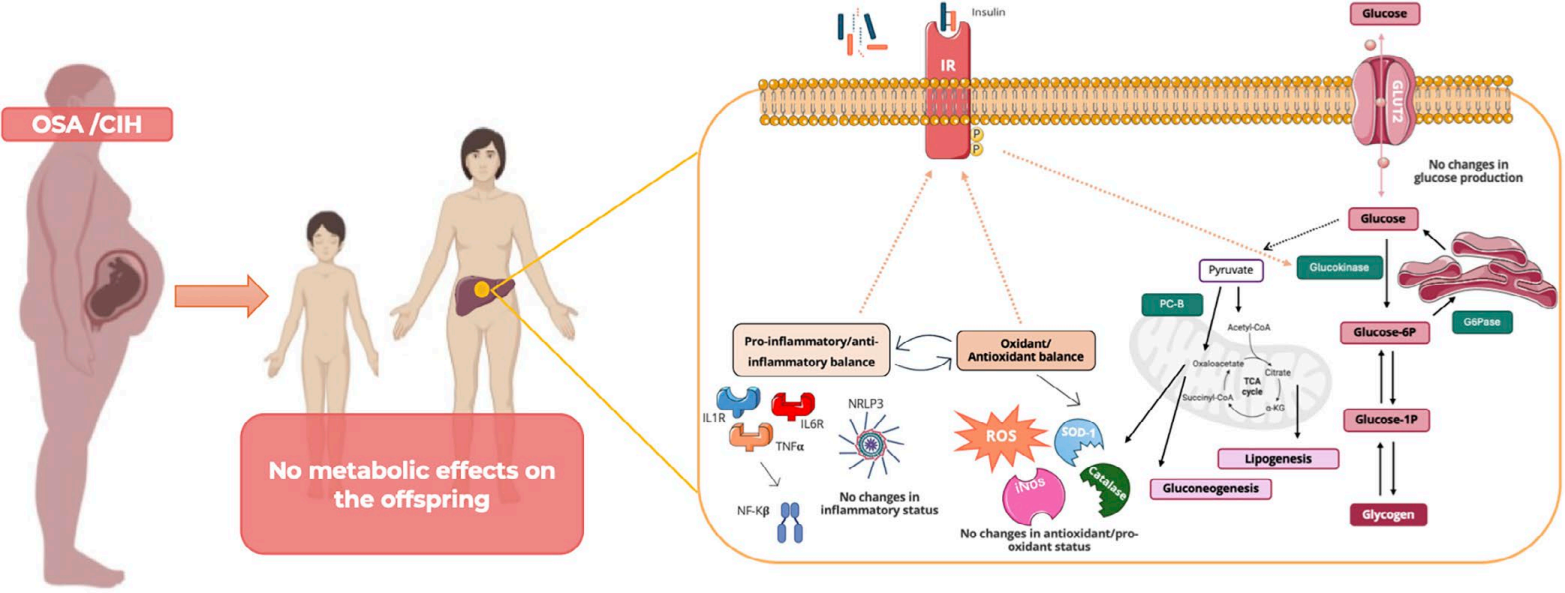
Schematic representation of the absence of effects of maternal OSA/CIH on metabolic phenotype and on key enzymes of hepatic inflammation, pro/antioxidant balance and glucose metabolism in the offspring. Glucose‐1P—glucose 1 phosphate; Glucose‐6P—glucose 6 phosphate; G6Pase—glucose‐6‐phosphatase; iNOS—inducible nitric oxide synthase; PC‐B—pyruvate carboxylase; SOD‐1—superoxide dismutase 1.

Herein, we found that maternal CIH did not affect offspring body weight, fasting glycaemia, glucose tolerance or insulin sensitivity at 1, 3 or 12 months of age. Additionally, gestational CIH had no impact on the hepatic expression of proteins involved in glucose metabolism, antioxidant defence and pro‐oxidant and inflammatory pathways at any of the offspring ages studied (Figure 6). As expected, biological ageing induced changes in whole‐body metabolism, antioxidant capacity and inflammation; however, these were not influenced by gestational CIH (Figure 6). No sex‐dependent differences were observed.

These findings suggest that 2 weeks' gestational exposure to CIH—mimicking OSA during pregnancy—may not be sufficient to induce long‐term metabolic dysfunction in the offspring. This may reflect a protective role of the mother against the deleterious effects of CIH.

Previous studies have reported that gestational exposure to CIH or OSA can negatively impact offspring body weight and metabolism, and particularly when the exposure is prolonged or combined with other insults such as maternal obesity or a high‐fat diet (Badran, Yassin, et al. 2019; Farabi et al. 2021; Iqbal and Ciriello 2013; Khalyfa et al. 2017; Sanapo et al. 2024; Telerant et al. 2018). Gestational CIH in animal models has shown in male offspring, increased body weight, impaired glucose tolerance, enhanced adiposity, and insulin resistance (Iqbal and Ciriello 2013; Khalyfa et al. 2017; Badran, Yassin, et al. 2019). In contrast, our data show that the gestational CIH paradigm tested here did not induce any detectable metabolic alterations in either male or female offspring. These differences in the studies may be explained by variations in the duration and intensity of CIH, the gestational window affected, or protective mechanisms operating in the mother or foetus. However, it is important to note that in our study we have used a moderate to severe paradigm of CIH, composed of cycles of 20% to 5% O_2_, that would be expected to cause a more deleterious phenotype (Wang et al. 2018). It is also important to note that human OSA prevalence is generally lower in the first trimester (3.6%–10.5%), increases in the second trimester (8.3%), and can become quite high in the third trimester, potentially reaching up to 27% in high‐risk women (Maniaci et al. 2024). Although our paradigm of CIH exposure during the final 2 weeks of pregnancy mimics OSA during human pregnancy, it may have allowed sufficient time for compensatory responses to occur and prevent long‐term programming effects in the offspring. Experimental factors such as animal strain may also contribute to the differences observed across studies, as most of the previous studies were conducted in mice (Khalyfa et al. 2017; Badran, Yassin, et al. 2019). Another plausible explanation for the absence of long‐term metabolic effects in our model is the activation of maternal protective mechanisms that buffer the foetus against environmental insults such as CIH. The placenta plays a key role in this protection, acting as both a physical and biochemical barrier that regulates nutrient, oxygen, and hormone transfer between mother and foetus. Placental adaptations to hypoxic conditions—such as alterations in vascularisation, changes in transporter expression, or modulation of antioxidant pathways (Sferruzzi‐Perri et al. 2023; Ahrens and Singer 2025) —may mitigate the impact of intermittent hypoxia on foetal development. In fact, using the same gestational CIH rat model it was already shown that placentas from GIH‐exposed mothers showed an increase in foetal capillary branching, expansion of maternal blood spaces, and the number of cells of the external trophectoderm in the tissues (Valverde‐Pérez et al. 2023). Moreover, the placentas from gestational CIH rats (Valverde‐Pérez et al. 2023) and mice (Badran, Abuyassin, et al. 2019) were enlarged. However, placentas from gestational CIH mice showed growth restriction, impairments in placental angiogenesis and vascular remodelling (Wang et al. 2018), as well as hypoxia and oxidative stress (Badran, Abuyassin, et al. 2019), suggesting that not all species' placentas may be protective against gestational hypoxia insults. In addition, maternal metabolic and hormonal adjustments during pregnancy may also contribute to preserving foetal homeostasis under stress. Supporting this, Almendros et al. (2019) demonstrated, in sheep, that the placenta functions as an oxygen transfer buffer, damping the amplitude of maternal hypoxia‐reoxygenation cycles caused by obstructive apneas. Consequently, oxygen fluctuations in the umbilical vein were significantly attenuated compared to those observed in maternal arterial blood, reinforcing the notion that placental function may play a critical role in shielding the foetus from the full extent of maternal intermittent hypoxia. In addition, maternal metabolic and hormonal adjustments during pregnancy may also contribute to preserving foetal homeostasis under stress.

Given the central role of the liver in systemic glucose homeostasis, and since previous studies have reported increased body weight gain and metabolic dysfunction in male offspring of mothers exposed to CIH (Iqbal and Ciriello 2013; Khalyfa et al. 2017; Badran, Yassin, et al. 2019), we investigated whether gestational exposure to CIH affects hepatic glucose metabolism in the offspring. Although we did not observe significant alterations in whole‐body glucose metabolism at any of the ages studied—except for an increase in insulin resistance in males driven by higher insulinemia while basal glycaemia remained unchanged, we hypothesised that early hepatic biochemical changes might precede systemic manifestations. However, our analysis of PC‐B, glucokinase, and G6Pase, revealed no significant differences between CIH‐exposed and control offspring, suggesting that gestational CIH does not impact hepatic glucose metabolism, at least at the level of these enzymes. In line with these findings and considering previous evidence linking OSA to hepatic oxidative stress, inflammation, and liver disease (Almendros et al. 2022; Aron‐Wisnewsky et al. 2012), as well as reports associating CIH‐induced dysmetabolism with similar hepatic disturbances (Fernandes et al. 2023; Savransky et al. 2007), we further assessed markers of antioxidant capacity and oxidative stress and inflammation in the liver. Our results showed that gestational CIH did not affect hepatic antioxidant defences or pro‐oxidant and inflammatory markers, reinforcing the idea that the liver's redox and inflammatory status remains stable under our experimental conditions.

Although gestational CIH did not affect hepatic oxidative stress markers, we observed an age‐dependent increase in SOD‐1 expression, which is in line with the existing literature (Gong et al. 1997; Hussain et al. 1995; Rikans et al. 1992). This finding suggests an adaptive antioxidant response in the ageing liver, possibly as a countermeasure to increased reactive oxygen species (ROS) production, that is known to be a feature of biological ageing (Anantharaju et al. 2002). In parallel with the oxidative changes found herein, offspring ageing was also associated with increased basal levels of inflammatory markers such as IL‐1 and TNF‐α receptors (Figure 5), indicating a gradual shift toward a pro‐inflammatory hepatic profile. This is consistent with the concept of ‘inflamm‐ageing’, which describes ageing as a chronic, low‐grade inflammatory state which is characterised by elevated levels of pro‐inflammatory cytokines (e.g., IL‐1, IL‐6, TNF‐α), chemokines, and acute‐phase proteins, alongside declining levels of anti‐inflammatory and antioxidant defences (Rea et al. 2018). Our data clearly align with previous findings in aged rodents, where increased hepatic levels of IL‐1β, TNF‐α, and chemokines have been reported (Anantharaju et al. 2002; Jin et al. 2020; Meng et al. 2023). However, we did not measure anti‐inflammatory markers such as IL‐10, which play a crucial role in maintaining immune homeostasis (Anantharaju et al. 2002; Jin et al. 2020; Meng et al. 2023). Nevertheless, altogether, our findings indicate that biological ageing drives oxidative and inflammatory changes in the liver, regardless of gestational CIH exposure. While these results suggest that gestational CIH alone does not induce overt basal immune alterations in the offspring, we acknowledge that additional studies assessing activation markers (e.g., cytokine secretion profiles, phosphorylation states) are required to determine whether latent or stress‐induced immune dysregulation may occur.

In conclusion, our findings suggest that 2 weeks exposure to gestational CIH, mimicking moderate to severe or undiagnosed OSA in late pregnancy, may not be sufficient to induce long‐term metabolic or hepatic dysfunction in the offspring, nor alter biological ageing, regardless of sex (Figure 6). This could offer some reassurance for women with OSA during pregnancy. However, caution is warranted, as our study focused on a specific window and severity of CIH. More severe or prolonged exposures—especially when combined with other risk factors such as maternal obesity or high‐fat diet—may still pose significant risks. Future studies are essential to thoroughly investigate these scenarios and better define the limits of foetal resilience to gestational hypoxic insults.

## Author Contributions

The authors have contributed to the study as follows. Participated in research design, A.R, S.V.C.; conducted experiments, E.V.‐P., M.B.A., J.F.S., E.O., J.P.L; performed collection and data analysis, E.V.‐P., M.B.A., J.F.S., E.O., J.P.L, S.V.C.; wrote or contributed to the original writing of the manuscript, E.V.‐P., S.V.C; contributed to the review and editing of the manuscript, E.V.‐P., M.B.A., J.F.S., E.O., J.P.L, A.R., S.V.C. All the authors have approved the final version of the manuscript and agree to be accountable for all aspects of the work in ensuring that questions related to the accuracy or integrity of any part of the work are appropriately investigated and resolved. All authors have read and agreed to the published version of the manuscript.

## Conflicts of Interest

The authors declare no conflicts of interest.

## Supporting information


**FIGURE S1:** (A) Custom‐designed intermittent hypoxia chamber for rodents. The octagonal chamber is divided, with detachable walls, into eight triangular compartments connected to a central distribution column with holes to ensure homogeneous gas delivery. The schematic on the right shows the detachable lid with handles that facilitate safe manipulation for opening and closing the chamber. (B) Schematic representation of the control interface for the intermittent hypoxia system. The setup includes a nitrogen compressor (upper panel, labelled as NITROGEN) connected through an electronically controlled valve (EV1), and a fan‐assisted air supply unit (EV2 and EV3; not shown in the picture as they are enclosed within the equipment). The gases are introduced through a distribution cylinder into an octogonal chamber with 8 allocated spaces for animal study. The system allows automated cycles between nitrogen and room‐air delivery with real‐time monitoring of valve activity (EV1–EV3), number of cycles, and gas distribution commands. Oxygen concentration is continuously recorded using an oxymeter (Oxydig, Dräger, Germany) and displayed in the lower graph.

## Data Availability

The data that support the findings of this study are available from the corresponding author upon reasonable request.
