# Supplementary material for: Exposure to Gestational Intermittent Hypoxia Does Not Impair the Metabolic Function or Accelerate the Biological Ageing Process of Offspring of Either Sex
Source: J Sleep Res. 2025 Nov 14;35(3):e70245. doi: 10.1111/jsr.70245 (PMC13193380; doi:10.1111/jsr.70245)
Supplement: Supplementary file 1 — FIGURE S1: (A) Custom‐designed intermittent hypoxia chamber for rodents. The octagonal chamber is divided, with detachable walls, into eight triangular compartments connected to a central distribution column with holes to ensure homogeneous gas delivery. The schematic on the right shows the detachable lid with handles that facilitate safe manipulation for opening and closing the chamber. (B) Schematic representation of the control interface for the intermittent hypoxia system. The setup includes a nitrogen compressor (upper panel, labelled as NITROGEN) connected through an electronically controlled valve (EV1), and a fan‐assisted air supply unit (EV2 and EV3; not shown in the picture as they are enclosed within the equipment). The gases are introduced through a distribution cylinder into an octogonal chamber with 8 allocated spaces for animal study. The system allows automated cycles between nitrogen and room‐air delivery with real‐time monitoring of valve activity (EV1–EV3), number of cycles, and gas distribution commands. Oxygen concentration is continuously recorded using an oxymeter (Oxydig, Dräger, Germany) and displayed in the lower graph. [file JSR-35-e70245-s001.pdf]

A

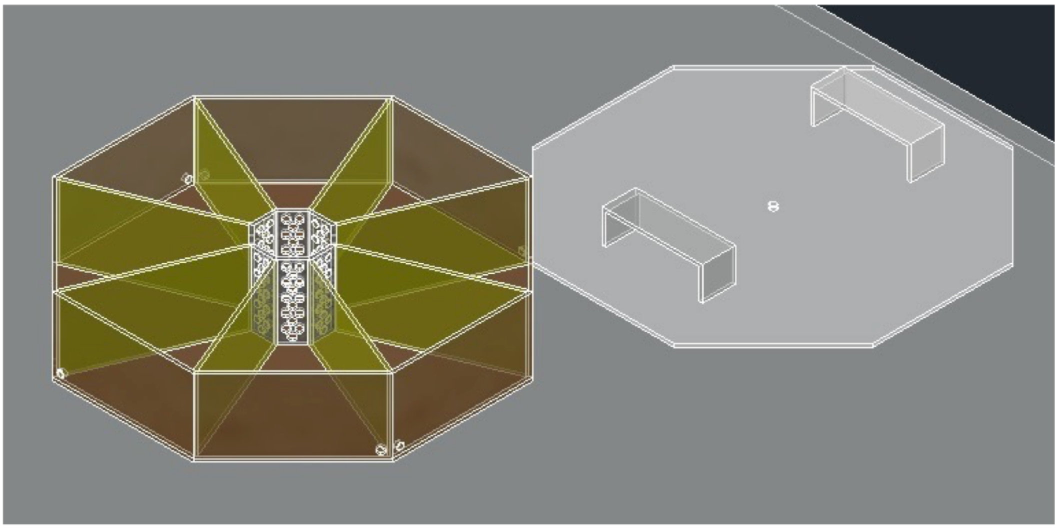

B

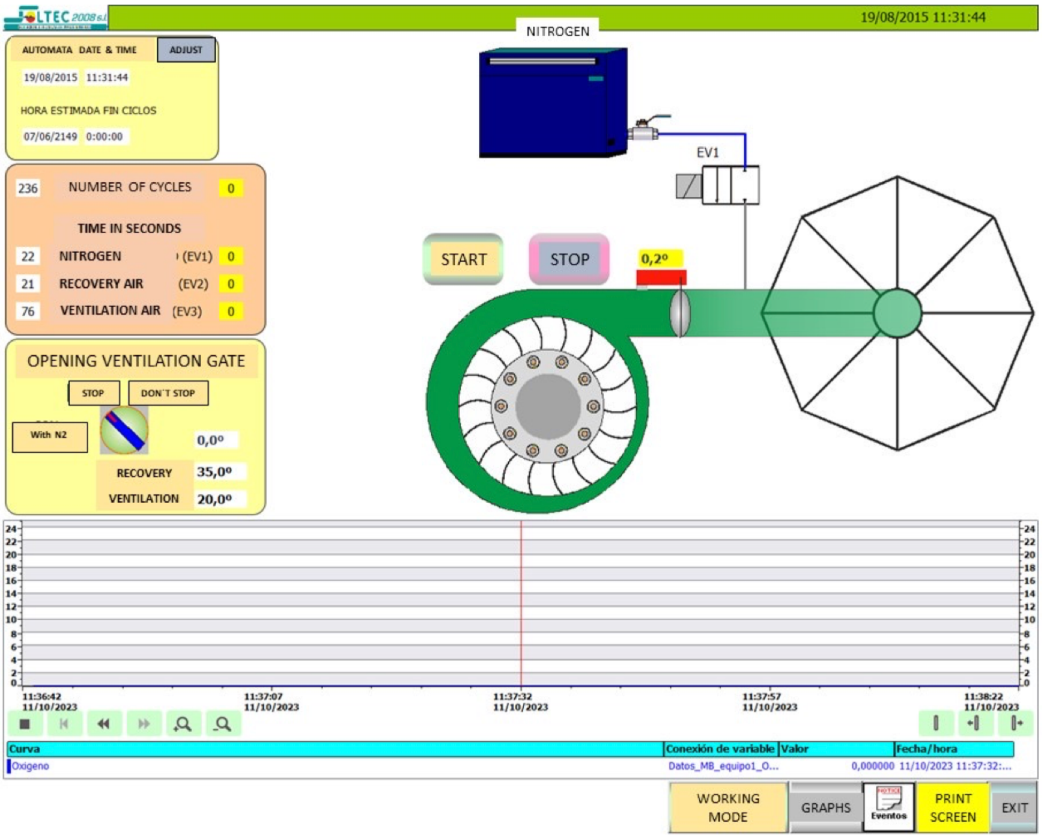

Supplemental Figure 1. A) Custom-designed intermittent hypoxia chamber for rodents. The octagonal chamber is divided, with detachable walls, into eight triangular compartments connected to a central distribution column with holes to ensure homogeneous gas delivery. The schematic on the right shows the detachable lid with handles that facilitate safe manipulation for opening and closing the chamber. **B)** Schematic representation of the control interface for the intermittent hypoxia system. The setup includes a nitrogen compressor (upper panel, labeled as *NITROGEN*) connected through an electronically controlled

valve (EV1), and a fan-assisted air supply unit (EV2 and EV3; not shown in the picture as they are enclosed within the equipment). The gases are introduced through a distribution cylinder into an octogonal chamber with 8 allocated spaces for animal study. The system allows automated cycles between nitrogen and room-air delivery with real-time monitoring of valve activity (EV1–EV3), number of cycles, and gas distribution commands. Oxygen concentration is continuously recorded using an oxymeter (Oxydig, Dräger, Germany) and displayed in the lower graph.
